# Chewing the fat on an emerging target in multiple sclerosis

**DOI:** 10.1016/j.ebiom.2021.103603

**Published:** 2021-10-02

**Authors:** Chai K. Lim

**Affiliations:** Macquarie Medical School, Macquarie University, 75 Talavera Road, North Ryde, NSW 2109, Australia

In this article of EBioMedicine, An Cheng and colleagues show that blocking the function of fatty acid-binding proteins (FABPs) pharmacologically, alleviates the severity of the inflammatory demyelinating neurological condition in the experimental autoimmune encephalomyelitis (EAE) mouse model [1]. Suggesting that targeting lipid metabolism may be a viable therapeutic strategy against several pathological hallmarks of multiple sclerosis (MS). To appreciate this finding, we need to examine the role of FABP and its relationship with MS.

MS is a multifactorial autoimmune and neurodegenerative condition. The presence of proinflammation brought about by reactive T cells, microglia and astrocytes, oxidative stress, and mitochondrial dysfunction that perpetuate degeneration of neurons and glial cells, including oligodendrocytes, are common hallmarks of early MS. Hence, therapeutic targets that suppress proinflammatory activities by the immune cells and astrocytes and promote the remyelination capability of oligodendrocytes are priorities in MS research.

Fatty acids are known to be involved in the inflammatory response, oxidative stress, and mitochondrial dysfunction (all processes relevant to MS). Moreover, myelin (a lipid-rich membrane structure) production is dependent on fatty acid synthesis made by oligodendrocytes [2]. This makes lipid metabolism modulation a key therapeutic target considering increased adiposity is a risk factor and linked to increased disability in MS. Some have postulated that inflammation in MS is a secondary consequence of dysregulated lipid metabolism.

FABPs are lipid chaperones that regulate of fatty acid metabolism with differential subtype expressions that confer tissue and cell specificity. For example, the brain FABP (FABP7) is expressed by astrocytic cells while epidermal FABP (FABP5) is expressed in immune cells such as microglia. There is early, but limited, clinical evidence showing that FABP is involved in MS. A recent paper showed that increased FABP4 is associated with higher disability in MS patients [3]. Suggesting FABP modulation could be an attractive therapeutic approach, with the potential for and cell specific interventions in MS; the development of FABP inhibitors is currently underway.

Several studies targeting FABP to limit proinflammation and injuries to the central nervous system have demonstrated overlapping pathological hallmarks across neurodegenerative diseases including MS. For example, mice lacking FABP4 and 5 have reduced EAE severity associated with impaired proinflammatory activities [4]. A similar outcome can be achieved pharmacologically by a selective inhibitor of FABP5 known as EI-03 [5]. These results are still in their infancy and involve animal models with a primary focus on immune modulation.

Cheng and colleagues show that MF6, a novel ligand which binds FAB5 and 7, reduces EAE severity and attenuates proinflammatory activities by limiting reactive astrocytes consistent with previous findings [6]. The authors go on to provide direct evidence that FABP5 and 7 inhibition limit oxidative stress-induced damage in EAE by targeting reactive microglia and protecting oligodendrocytes; these are considered as key strategies in limiting MS progression. MF6 could be an ideal drug candidate to specifically target immune cells and astrocytes relevant to MS pathology.

Several questions remain. It is tempting, at first, to think that inhibiting lipid metabolism may be key to limiting MS progression or at least in the EAE model. However not all fatty acids are increased in MS as summarized in a recent systematic review [7] and some were decreased but beneficial to MS such as oleic acid [8]. This poses a contradictory predicament underlying the therapeutic mechanism of lipid metabolism inhibition from a basic research perspective, that is, using an all-or-nothing approach that creates the epitome of a double-edged sword: Does inhibiting FABPs also inhibit other beneficial fatty acids in MS? Are there potential side-effects from long-term use? Is there a need for multimodal treatment?

Although MF6 has been shown to be well-tolerated in an animal study, other factors need to be considered. A recent epigenome-wide study showed that narcolepsy, a sleep disorder, and MS had overlapping DNA methylation in FABP7 suggesting that its downregulation (or inhibition) may impact sleep physiology [9], which is a prominent problem in the MS community. While there are potential issues that need to be addressed, it also presents other opportunities. It is noteworthy to mention that inhibition of FABPs can increase cellular endocannabinoid anandamide levels [10] that can have protective effects against MS. Hence, inhibition of FABPs may be a promising target that is not limited to fatty acid metabolism.

The feasibility of modulating complex fatty acid metabolic processes as a multitarget treatment of neuro-inflammatory and -degenerative conditions such as MS requires precise mechanistic control including disease timing, cell specificity, and host fatty acid metabolome profiles to achieve a holistic precision medicine approach. The findings presented in this study make even more of a case for targeting multiple cell types responsible for proinflammation, oxidative stress, mitochondrial dysfunction, and myelination. This signals an exciting time in the next generation of treatments targeting FABP in MS and possibly in other neurodegenerative diseases that share some overlapping pathologies.

## Contributors

CKL wrote and reviewed the commentary and approved the final version.

## Declaration of Competing Interest

The author declares no conflict of interest.
